# Public perspectives on inequality and mental health: A peer research study

**DOI:** 10.1111/hex.13868

**Published:** 2023-10-02

**Authors:** Vanessa Pinfold, Rose Thompson, Alex Lewington, Gillian Samuel, Sandra Jayacodi, Oliver Jones, Ami Vadgama, Achille Crawford, Laura E. Fischer, Jennifer Dykxhoorn, Judi Kidger, Emily J. Oliver, Fiona Duncan, Members of the SPHR Public Mental Health Programme

**Affiliations:** ^1^ The McPin Foundation London UK; ^2^ Traumascapes London UK; ^3^ Division of Psychiatry University College London London UK; ^4^ Population Health Sciences University of Bristol Bristol UK; ^5^ Population Health Sciences University of Newcastle Newcastle United Kingdom

**Keywords:** mental health, peer research, photovoice, poverty, racism, structural inequality

## Abstract

**Introduction:**

Associations between structural inequalities and health are well established. However, there is limited work examining this link in relation to mental health, or that centres public perspectives. This study explores people's experience and sense‐making of inequality in their daily lives, with particular consideration of impacts on mental health.

**Methods:**

We conducted a peer research study. Participants had to live in one of two London Boroughs and have an interest in inequalities and mental health. Using social media, newsletters, local organisations and our peer researchers' contacts, we recruited 30 participants who took photos representing their experience of inequality and discussed them during semi‐structured interviews. Data were analysed using reflexive thematic analysis.

**Results:**

Three themes were identified in this study: (1) inequalities are unjust, multilayered and intertwined with mental health. Accounts demonstrated a deep understanding of inequalities and their link to mental health outcomes, describing inequalities as ‘suffering’ and ‘not good for anyone’. Financial, housing, immigration and healthcare problems exacerbated poor mental health, with racism, gender‐based violence and job loss also contributing factors for both poor mental health and experiences of inequality; (2) inequalities exclude and have far‐reaching mental health consequences, impacting personal sense of belonging and perceived societal value and (3) moving forwards—addressing long‐standing inequality and poor public mental health necessitated coping and resilience strategies that are often unacknowledged and undervalued by support systems.

**Conclusion:**

Lived experience expertise was central in this study, creating an innovative methodological approach. To improve public mental health, we must address the everyday, painful structural inequalities experienced by many as commonplace and unfair. New policies and strategies must be found that involve communities, redistributing resources and power, building on a collective knowledge base, to coproduce actions combatting inequalities and improving population mental health.

**Patient or Public Contribution:**

This study was peer‐led, designed and carried out by researchers who had experiences of poor mental health. Six authors of the paper worked as peer researchers on this study.

## 1 INTRODUCTION

There has been extensive research investigating UK health inequalities, which are known to be driven by social inequalities such as poor housing, lack of education, and unemployment or poor‐quality work environments.^1^, ^2^ People with more social capital and financial wealth have better health outcomes; social and economic disadvantage damages health and wellbeing.^3^, ^4^ Geography matters for health and illness^5^; there are variations by region and neighbourhood, for example, in access to health care, air quality, food security and levels of social capital, which all impact health outcomes.^6^ Addressing health inequalities is political, asking questions of capitalism and approaches to social justice, with academics keen to acknowledge scholarly deficits and the complexity of presenting issues, thus encouraging further debate.^7^, ^8^, ^9^ A social determinant of health approach has been offered as a framework to address health inequalities.^10^, ^11^ Yet, despite extensive evidence and UK policy initiatives,^12^ we have been living long‐term with a sustained public health crisis, resulting in disparities in mortality and health, including mental health.^13^

Work on the social determinants of mental health^14^ has consistently shown the importance of systemic and structural factors in driving inequality and, critically, the socioeconomic disparities and policies that sustain these.^15^ There is a social gradient, with higher levels of income inequality being directly linked to increased prevalence of mental health problems.^16^ Indeed, a recent review of the social determinants of mental health identified 55 interconnected factors at four levels: individual, family, community and structural. Structural elements included climate change, population displacement and the welfare system.^17^ Both mental health problems and health inequalities are socially patterned, with sociological and social‐psychological theories offering explanations.^18^ Groups at risk of poorer mental health outcomes have been identified as females, those of younger ages, those experiencing financial insecurity and those subject to abuse/stigma because of marginalised ethnicity or sexual orientation.^19^ Focusing on children and young people, an in‐depth analysis of national policy relating to mental health highlights a lack of acknowledgement at national and local levels that mental health can cause inequalities.^20^ An increasing focus on this link is needed, particularly as the Covid‐19 pandemic brings to the fore growing inequalities^21^, ^22^ and increased mental health needs among specific groups,^23^ including rising demand for a range of mental health services and supports.^24^

Most work on health inequalities in the United Kingdom has adopted an epidemiological approach, providing strong evidence for policymakers to act.^25^, ^26^, ^27^ Complementing this are a few qualitative studies, including work in the Northwest of England.^28^ Public perspectives were collected to theorise health inequalities, with detailed knowledge emerging of the many diverse factors underpinning damaged health. Another study, in the Northeast of England, adopted an ethnographic approach identifying structural, material and psychosocial influences driving health inequalities. Fatalism and lack of control over circumstances were common reasons given for inequalities. Place‐based stigma was also highlighted.^29^ A meta‐ethnography using 17 studies produced a simplified model of socioeconomic health inequalities from lay accounts; when asked, the British public have a very good understanding of the links between hardship and ill health.^30^ However, these studies explored health in general, and tended to conceptualise it foremost as an outcome of inequalities. Building on this understanding, we extend this work in two important ways. First, we focus on mental health as our key concept of interest, recognising it impacts on peoples' lives in particular ways, when compared to physical health. Second, we centre on the perspectives of expert public members, both as researchers and as research participants, to provide rich and unique insights into how inequalities are experienced in relation to mental health.

## 2 METHODS

### 2.1 Research approach

This was a qualitative study using peer methods,^31^ a participatory approach where people affected by the issues being researched directly and conduct the study.^32^ It is a useful method for working closely with communities and individuals who can feel exploited and marginalised by research processes that lack cultural humility.^33^ Peer research benefits from insider knowledge and attempts to ensure an equilibrium of power between the interviewee and the interviewer.^34^ To aid this, here we drew on our personal experiences of poor mental health and inequality, including homelessness, race‐based discrimination and gender‐based violence.^35^ The peer research team were recruited originally to work on a public mental health research programme advising academic teams.^36^

The study was designed by a group of 5 peer researchers (L. F., O. J., G. S., A. V. and M. P.). We were inspired by photovoice,^37^ another participatory method. We incorporated an element of photo taking into the study but not the full photovoice method. It was delivered by six peer researchers and two research managers (G. S., A. V., O. J., A. L., S. J., A. C., V. P. and R.T.) and several academic advisors over a 16‐month period from May 2021 (J. D., E. O., J. K. and F. D.). Reflexivity was built into the research process throughout, both when considering and discussing potential biases and supporting team wellbeing.^38^ The team received training in trauma‐informed practice (by L. F.) before commencing interview work. Peer researchers' experiences ranged from no previous experience of qualitative research to having completed PhDs using qualitative methods.

### 2.2 Setting

This research was carried out in two London Boroughs (Harrow and Lambeth), selected because of established networks facilitating recruitment within their diverse communities.

### 2.3 Sample

We recruited through social media posts and newsletters, posters in local cafes, libraries or community centres, direct contact through local organisations, our peer researchers' own networks and word of mouth. The information sheet made clear that peer researchers working on the study all had their own experiences of poor mental health and outlined a voucher payment of £100 for taking part. Interpreters were offered (taken up by one participant) or advocates encouraged to attend interviews as sources of support (attended in two cases).

We interviewed 30 people from 56 enquiries. The aim was to explore public perspectives from a diverse group of participants, so predetermined inclusion criteria were kept to a minimum. Eight people were excluded as they did not live in Harrow or Lambeth. Seven people did not want to receive the information sheet after first contact, 10 declined to take part after reading the information sheet and 1 person could not find time to be interviewed.

We recorded the demographics of our participants and monitored these to ensure we targeted latter recruitment for maximum diversity, thus adopting a purposeful sample strategy. See Table 1 for the final sample.

**Table 1:** Demographic characteristics of participants (*n* = 30).

Gender	
Male	15
Female	13
Nonbinary	1
Prefer not to say	1
Age	
18–24	3
25–34	7
35–44	5
45–54	5
55–64	6
65 plus	3
Prefer not to say	1
Ethnicity	
Asian or Asian British—Indian or Asian other	8
Black or Black British—African or Black British Caribbean	8
Mixed—White and Asian/Asian British	1
Mixed—White and Black/Black British African or Caribbean	2
Mixed other	1
White British	3
White other	2
Other	2
Prefer not to say	3
Location	
Harrow	20
Lambeth	10

We also summarised our participants' mental health experiences into four groups (see Table 2). Each had unique experiences, over varying lengths of time, with depression and anxiety descriptions being the most common (cited by half).

**Table 2:** Mental health experiences among participants (*n* = 30).

Mental health experiences	Number of participants	Example quotations
Experience of mental health problems and receiving current treatments, often using diagnostic terms to describe.	17	Well, I've been through a lot of traumas in my life. I suffer from OCD and depression, and a lot of anxiety. I worry about things constantly, and even today. (08)
		It's borderline personality disorder, but over the years there's been several different diagnoses, so I'm not sort of like a hundred percent sure, but they seem to have settled on that one for now. (11)
		I think for me, it is kind of the route of, like, other mental health conditions. Like, I would say, like, autism is the little stem and then out of that comes, like, depression and anxiety, etc., etc., OCD, and this and that, over the years. (07)
Experience of mental health problems—sometimes described with a diagnostic label but not always and no treatments explained. Some people in this group had severe periods of mental health issues but short term, others suggest longer‐term problems or episodic challenges.	10	I started getting these mental health problems in sense of depression, I suffered a lot from depression at one time. (05)
		I stay there [at home] when I feel depressed. It's the only way that I can relax and I can… it's my windows room, you know. I stay there and look at the tree. What is important is there is activity in this tree. (13)
		Oh loads, throughout uni, I suffered from really bad chronic depression, just mental ailments, just not feeling 100% myself, not feeling like I wanted to leave my room, very just inwards which is, for me, it was a long process, it took me ten years to really manage that and I would figure out why and my motivations and how to look after yourself and things that you should probably be taught at school a bit more, how to manage your own mental health really and the things that make you tick. (27)
Experiencing poor mental health (short term) linked to the Covid pandemic, no diagnostic labels, no prescribed treatments described.	2	I didn't work from 2020, 2021. So, that was quite difficult on my mental health because you do doubt yourself a little bit. And I was thinking, ‘Am I good enough? Do I need to get back on this – how do I – yes, how do I get back to where I was?’ It's difficult. It is difficult. (29)
No personal mental health problems were disclosed but experience as a carer.	1	I had to leave my job, to help my daughter at the hospital. In mental health, having a child with mental health issues, it's not easy, especially when they are admitted to hospital. It was an awful experience for the whole family. (16)

Abbreviation: OCD, obsessive–compulsive disorder.

### 2.4 Procedures

Following consent‐taking, participants were offered disposable cameras, although most preferred to use their own phones (*n* = 26), to take 3–4 photos depicting inequality. We gave minimal guidance; the photo‐taking process was to provide a starting point for the interviews of things participants wanted to share to explore what inequality meant to them. Two participants created a video. Photos and videos were shared with peer researchers before the interviews. We conducted interviews using participant's preferred format: in‐person (*n* = 12), via Zoom (*n* = 14) or by phone (*n* = 3).

We interviewed in pairs, with the conversation audio‐recorded. The most experienced interviewer and project coordinator (A. L.) was in every interview alongside G. S. (*n* = 4), S. J. (*n* = 13), A. C. (*n* = 4), O. J. (*n* = 5) and A. V. (*n* = 2). There were two exceptions, where participants requested only A. L. be present. Peer researchers did not interview participants they knew, except in one requested case. Peer researchers were allocated interviews based upon their availability but also shared identity and personal experiences, including gender, ethnicity, age and mental health diagnosis.

The interview schedule was amended after 10 interviews following the initial data review (see Supporting Information: Appendix 1). The change was to explore mental health experiences further and five participants were reinterviewed. Peer researchers connected with participants by sharing their own experiences appropriately, building empathy in interviews, helping people feel at ease and acknowledging the impact of trauma.

Peer researchers debriefed after each interview in pairs, and a manager was also available. Reflective notes postinterview recorded personal reflections, including where information shared resonated with own experiences. An example is provided from one peer researcher.

The interview was underway; I felt a stirring inside me; when she spoke of inequality within her culture, this resonated with me; also, disparity of wealth, another struggle both external and from within.

Being treated unfairly through childhood and beyond was her norm, as it was for me. A stirring rage inside, drives her forward to a better life, self‐made, like mine. I came to realise that applying my ‘peerness’ (lived experience) to an interview is not a practice learned. It's a feeling.

The trick is to allow my internal feelings of understanding and empathy guide my responses. Then, and only then, articulate with elegance, hoping that the words to questions not yet formulated in my head would reveal themselves, which they did, somehow, to advance the interview appropriately.

How fortunate I am to have suffered and then survived.

### 2.5 Analysis

We conducted an inductive collective thematic analysis drawing upon published principles for reflexivity^39^ and for coding.^40^ An external transcription company produced transcripts which were checked for accuracy. Data familiarisation involved the entire team, with ideas about key experiences within participants' data discussed in team meetings after the completion of 10 interviews. A list of pattern ‘codes’ was generated by the team, which were revised and adapted as we reviewed additional data. Six transcripts were selected to help us build a detailed code list to use in Excel (see Supporting Information: Appendix 2). For all 30 transcripts, relevant text relating to the inequality factor being discussed, its context, manifestation and impact were captured, with a focus also on how participants defined inequalities, mental health and the perceived links between them. Once all data had been coded, we held a workshop to move from codes to themes, with discussion drawing from researchers' own lived experiences to make sense of how details could be framed as broader cross‐cutting ideas. These themes were then reviewed using an iterative process revisiting data; the final three themes are presented below with illustrative quotations.

## 3 RESULTS

### 3.1 Inequalities are unjust, multilayered and intertwined with mental health

Our participants had clearly developed views around what inequality was, providing both personal and technical responses. Technical definitions included referencing^41^ and wider determinants of health framework. Inequality as lack of access to opportunities or differences in opportunities available to people, because of financial circumstances or personal characteristics such as race or gender, was a common thread. It was also described as ‘everyday life’.

It's everyday life for people, isn't it? It affects people in different ways. […] In every way, with housing, with benefits, with everything. (20)

Inequality was ‘suffering’, and it was acknowledged that inequalities were ‘not good for anyone’ because of the societal tensions they create.

Inequality is illness, inequality is social unrest, inequality is spread out in many, many, many different ways and the rich getting richer, the poor getting poorer and that's not good for anyone. (06)

That is broad. I suppose it means, like, the picture I get is like hardship, and struggle and pain and things like that. I think, for me, it means, sort of lots of bad things that happen in the world. […] People that suffer from it, so yeah, suffering in one word, maybe. (18)

Inequality exists in every aspect of our life. Whether you have mental health problems or not. I think when you have mental health problems, they are magnified. (10)

These conversations consistently linked inequality with mental health experiences, ‘they're just tied up in each other’, and acknowledged that health problems and disability themselves create an increased likelihood that inequalities will be experienced. The public perspective is one of agreement when asked about any links between inequalities and mental health: ‘I think there is 100% yes’ (25). Across the accounts, we heard that people experienced many challenges and system barriers to access help, information and support, rather than a single issue. When people are dealing with ‘a whole ton of issues’, the mental health impacts can be more extreme and difficult. These experiences were also commonly underpinned and exacerbated by wealth disparities.

I think inequality just exacerbates mental health problems, and a lot of the time, they're just tied up in each other. […] All my mental health issues I feel are all the stuff I went through when I was younger. It's stuff that happened in my family that can definitely happen, you can still get abusive relationships and substance abuse disorders in people who are more well off or in a higher class, but when those situations arise in a working class or immigrant context it's even harder to deal with, because we're already dealing with a whole ton of issues. We just end up getting the worst of it because we don't have the means to get the help that we need, we can't afford to send relatives to rehab, or afford the appropriate therapy for someone. (22)

Inequalities were also described as contributing to or causing traumatic life events that prompt help‐seeking for mental health issues, not necessarily at the time but years later. ‘Growing up’ in adversity, managing gender‐based violence in the home is a human rights issue.

Yes, I mean, my mental health, started gradually. I mean it was in different patches. I mean, when I was young, I was abused because I was a one parent family. My mum used to take me to brothers. The children of the brother, really, they did not like me. And I was moved from one place to the other. So that I think affected me when I was growing up. (09)

Personal experiences of inequality can be traumatic: ‘Trauma is a very big thing’ (4). Participants explained how the strain of living with inequalities contributes to poor mental health, and how living with a mental health diagnosis, and associated public and internalised stigma, was associated with increased likelihood of having to deal with difficult life outcomes. We provide three examples (see Table 3) with extracts from interviews unpicking these interactions. A central theme was the loss of opportunity that both experiences of inequality and poor mental health impose. Poor access to support systems because of structural and process barriers makes it harder for those in need to navigate the ‘ivory tower’.

**Table 3:** Exploring inequality and mental health.

Inequality in systems	Impacts	Reference to inequality and mental health
Immigration services—being an immigrant, struggle to understand UK systems in nonhome language Housing departments (Local Council)—including homelessness Economy—living in extreme poverty Employment—being less able to work and find work Healthcare services—lack of access	Loss of income Housing and local environmental Healthcare Loss of opportunity Trauma and mental illness Other health‐related problems	I have this child, I had to struggle all my life and then as well coming to a foreign country, you have to start from zero really because the fact of this is not my native language, it's kind of a disability. It takes you all the effort to understand things and then building my case, **I did have very traumatic experiences, it's affecting my mental health, having bad housing in this country with the cold weather, it's affecting my health as well, my weight and not able to have a full‐time job because my health deteriorated very badly**. It makes me live in extreme poverty today and resorted in having to live in a homeless hostel when **I did have a…managed to get a house here and have a job, but I managed to lose it all really because of illness**, because of unlucky when this happens and not be able to probably understand the system that there were benefits that would have helped me get back on my feet sooner rather than later, so I have experienced suffering that probably I didn't need to because **if I had the right information, the right people supporting me, probably I could have managed to get my situation back together and okay. Get my health sorted sooner and be able to achieve more**. (06)
Economy—being lower socioeconomic status Immigration services—dealing with the immigration system Lack of access to services—as an immigrant	Loss of income Loss of trust in institutions or support systems Loss of opportunity Trauma and detriment to mental health	Times I was unwell the root of this discomfort were at things personal and wider circumstances I was in connected with inequality whether a socioeconomic status or fewer opportunities in life to access services to trust service providers sometimes even under the new rules of immigration to be recognised as a subject allowed access certain services. There is often that feeling of an ivory tower whether it is in talk or in preventing me from engaging with larger institutions with larger structures of the society that make the hurdle higher and **it's not easy to overcome it. Continuous fight is exhausting, consuming lots of energy that is precious in other aspects of life and a series of failures to overcome these hurdles is detrimental to, leaves you with a trauma, leaves you with that numbness, that feeling of being numb, giving up**. (01)
Racism in society Workplace discrimination	Loss of opportunity Mental health issues General health deteriorates	Right, basically the link is when **people are treated so badly with inequality it doesn't go away, that treatment of the way they're treated as a huge impact on their mind which then has a huge impact on their wellbeing and their health**. It's not just all about mental wellbeing it's their general health that starts to suffer as well. I mean I have known [people] which have not been treated properly and have had mental health issues and as a result of the mental health issues they have actually had other effects and they've caught diabetes as a result of their stress, so all of that sort of stuff yes. […] Well certainly as a child when we faced racism in Liverpool being called a ‘Paki’ I didn't know what a Paki was, always having your windows smashed in and then the words NF written and sprayed on your door and we've also had ‘Wogs out’ obviously, I don't know why they call me a Paki, I'm from India, […]. But they class us as one because of the colour of our skin, […] **That has a huge impact, racism has a huge impact but to have actually face [workplace] bullying is even worse and I don't know, again is it because of my race again, why was I picked out that way?** 1 min I'm getting a letter from the director saying well done next minute the line manager is telling me you're not doing well at your job, we're going to have to let you go, there's something not right. Again is it because I'm a male, I don't think so because there were other males there, and **I think it's because I'm Indian and it's because of my race and that's what caused it [being made redundant leading to severe depression]**. (24)

The accounts in Table 3 also evidence the complexity of living with inequality and poor mental health; multiple factors can feel inescapable. How ‘inequality doesn't go away’ providing unrelenting long‐term pressures that are compounded by racism, inaccessible support systems (health care, immigration services and welfare), housing problems and job loss. For example, we heard in relation to finding work, stable housing and health care (see participant 06 in Table 3), about the stress and strain that people lived under in these circumstances: ‘it was just unbearable’ and the ‘struggle’. People also acknowledged the frustrations of not being able ‘to achieve more’. Housing and health care are basic needs, but the systems involved, including the council and healthcare agencies, can be slow to respond and difficult to navigate. As participant 06 explained, their experience of large structures like the immigration service was of facing multiple barriers to secure help: ‘the continuous fight is exhausting’. We also learn about the ‘huge impact’ of racism both in neighbourhoods and in the workplace from participant 24. Racism and race‐related terms were discussed in most accounts, with 15 people recalling personal examples of discrimination related to being a person from a racialised community. It was acknowledged‘it's not easy to overcome’ (01) such disadvantage.

### 3.2 Inequalities are excluding with far‐reaching mental health consequences

The accounts describe the intense and all‐encompassing consequences of having to deal with inequalities. Making sense of these experiences, we focus on two areas: sense of belonging and societal value (see Table 4). These were developed to illustrate challenges for addressing inequalities and poor mental health.

**Table 4:** Exploring the consequences of inequalities and poor mental health.

Theme	Examples	Extract
Personal: sense of belonging	Shifting identity from professional role as a midwife to managing mental health problems. Exploring previous held stigma—them‐and‐us beliefs. Gentrification and leaving Black‐owned businesses to struggle and close changing the character of the area and people's connection to place (Lambeth) and commitment to each other. Race‐based discrimination, moving from one region to another and experience problem with the sense of belonging that impacted mental health.	And as a result of that my mental health suffered, and that's when I got known to the mental health system. But prior to that, because I was seen as, in my eyes I was a professional, doing well, I didn't think that I could have mental health problems. So when it happened and I was brought into the mental health system, I realised actually, it took me a while to realise, but I thought, in a sense, it was a case of them and us. So like, people's mental health problems were them, and then there was me and everybody else. But when I got into the system, I realised I'm part of the whole thing anyway. (23) If you went back to [name of area], to bring it back to mental health, 20 years ago, people walking through there would be like, ‘Hi, you alright? You good? What's going on? How's your kids? Blah, blah’. Now, you walk through there, it's just, ‘I need to go here. I need to get where I'm going. I don't care about any of you people, just move out of my way’. Gentrification and all this money being injected has definitely, I think, affected people's mental health, especially in a city like London where it's so fast‐paced anyway that people are not really caring about other people, and what they're going through.[…] I hate this shop but I'm spending my money there. I'd say is, I just want more togetherness, I'd say. Yes, and I guess belonging, I'd say. I guess belonging. (29) That was 1972, it got so bad we had no choice but to leave Liverpool to come to London because guess why we come to London because there were a bit more brown and black people and ethnic minority people so that means we might be getting support from others. Because we're the only brown people in Liverpool at that time and it was strange, hey you know everyone else is white, English, what are you doing in our country you don't belong here. And since then I presume that's what's caused the mental health issues ever since that day, but we've done our best to overcome it […]coming to London we became a bit stronger but then coming to London we faced something in the late 70s, early 80s called the National Front and the more Indian people there were, the more ethnic minorities there were, the British people started to reject us and the British people started to get together themselves and they caused a movement called the National Front and now it doesn't exist but it's called BNP, so British National Party so unfortunately it's one of those things we've had to struggle with. (24)
Society and culture—how individuals understand how they are valued	Abuse experienced while living on the streets—public view of people who are homeless. Struggling to get life back on track, after losing a job and wife, and wanting things to get back on track, to have a role in society. Not feeling valued in university/youth dance environment, the experience of whitewashing impacting on mental health. Teacher experiences a lack of understanding in the workplace towards mental health problems and trauma.	Abuse. Yeah, this is what I get all the time, I get called a prostitute, a drug addict, ‘Get a life’. People say, ‘You can't be homeless, you're not dirty enough’. Just constant abuse, ‘Get a job’. People offering money for sex, saying, ‘Well, it's better than just sitting there’. It's like, ‘No, at least I can shut my eyes of a night and know [Laughs] that I haven't gone all the way down. […] People have perceptions, don't they, and they just believe that everybody[…] I had a woman the other day stop me and say, ‘I've been told that everybody who begs is a drug addict. (20) How can I do this, where shall I start, where shall I go, what is the best place to start? And how? And I'm not happy with the company, I want to, it's not just waiting for the money. It's the way they treat me. It's not fair. And with them I lose everything, I lose my wife, I lose my partner, I lose my job, I lose my confidence and I have problems. Now I have some, how shall I say, bills behind me. Because nothing was in the place Universal Credit was claiming, it take a long process. And it's a lot of mess. […]Yeah, now I'm with mental health, and I tell them I need some therapy groups. I want to get back into society and it's the only way for me, I think, to have talking therapy with a group. Before, I wanted just with one person, otherwise my confidence with one person is like now, being two, I'm happy. With one person it's alright but just, it's not enough. I want to be confident with people. I want to share my experience, not being ashamed and to understand that, all the time I've taken out, then some will understand you, I can step in. I can be out, take those steps and be out around the people, like, okay, someone will listen to me, maybe. One day, everything will come back. (19) So, I can give you examples of in choreography, in my university experience, if I came in with dance hall movements or afro movements or house movements, it was just like, ‘That's not what we're asking for’. It was a fight. […] Now I'm really trying to return to that Afro Caribbean diasporic movement. I'm really trying to return to it, really trying to find it and that is because my experience in those youth companies, of people who didn't train in those institutions that were so whitewashed or were more welcome or just didn't train at all and were just passionate dancers, I didn't feel welcome or able. Not necessarily welcome. I didn't feel able to be in that environment with them and I think that's the impact that it had on my mental health, as in me losing myself because I'm like, ‘Well I'm still one of them’, but I just didn't feel right with them. (21) Yes and also even when you're just trying to stay employed full time employers don't really seem to have the empathy and understanding that is needed when it comes to understanding why someone might not be able to handle certain things or, I don't know, it's just because I worked as a teacher and I just found the whole experience really alienating, being absent or not going into work was like the highest crime you could do and you were just expected to come in even if you were feeling like absolute crap, whether it was mentally or physically to be honest … I feel like there was too much scrutiny, or just a lack of understanding, they didn't really understand how to deal with someone who had symptoms and anxiety and they also just didn't know how to talk to someone who had been a victim of sexual assault. I think one of the first things the head teacher said to me about it was something along the lines of don't get yourself into any dangerous situations or something like, and it was very like victim‐blamey, and I was like this guy clearly has no idea what he's talking about. (22)

#### 3.2.1 Personal: Sense of belonging

Identity was a strong subtheme, with individuals describing how experiencing a lack of opportunities, lack of access to services, different forms of discrimination and mental health challenges shaped their sense of self and feelings of belonging (see Table 4 for examples). Interviewees spoke of the importance of inclusion and acceptance, and how the absence of that could ‘make one's identity more fragile, more vulnerable, less visible in the society, in the community’ (01). There were also other intersectional identity features in the accounts, particularly focused on gender inequalities, including gender‐based violence and some hidden personal characteristics such as being neurodivergent or from the lesbian, gay, bisexual, transgender, queer, intersex and asexual+(LGBTQIA+) community.

I think sometimes I do think if I wasn't who I am, if I wasn't born into this life, a woman of colour, who's fairly okay socioeconomically but not exactly the best, I think my life would have been very different. I mean, as it would be, right? Some of the experiences and some of the traumas that I've experienced are very specific to my identity. (02)

In one example, we heard from a midwife who was struggling for several years coming to terms with workplace discrimination and new challenges brought on by poor mental health. She explained:

So I would say my depression started after I lost the job, and trying to fight. And it was so evident that the racism and inequality and everything existed in my case. […] The lawyer said it was a straightforward win case, but I couldn't handle it because my mental health had gone so far down, you know, to a point of suicide. (23)

It was her first experience of poor mental health, and she described how it forced her to reassess ‘who’ gets mental health problems and which groups of people she felt that she belonged to.

If I'm being honest, I was looking at ‘these people’, as ‘these people’ have got mental health problems and why am I here with ‘these people’? It was about four weeks later, when people started talking about their life experiences, I thought that actually, I am ‘these people’. There were doctors there, there were social workers, there were nurses, all different sorts of people, […] And we were all in the same group. And it's only then that my mental health started to change, when I realised that I'm actually in the same circle. (23)

After her sister died, she made peace with herself, no longer resisting being a mental health service user: ‘I realised I'm part of the whole thing anyway’ (23). Her sense of belonging shifted, and she embraced voluntary work with a local mental health charity, but it had taken over 4 years to get to a place of acceptance for what was lost, a valued career in nursing, and the trauma of dealing with racism in the workplace.

Individuals shared other examples of how their sense of belonging could be disrupted, or even prevented, by experiences of place‐based race discrimination:

What are you doing in our country you don't belong here. And since then I presume that's what's caused the mental health issues ever since that day, but we've done our best to overcome it. (24)

Other accounts focused on how the character of entire neighbourhoods and communities could be changed quite suddenly by the influx of money and gentrification, leaving those who remembered what had been there before with a sense of loss:

I hate this shop but I'm spending my money there. The way it affects me, I'd say is, I just want more togetherness, I'd say. Yes, and I guess belonging, I'd say. I guess belonging. […] I can go out and see my friends at these kind of places, these bars, these pubs, whatever, but we need these places for everybody. That's the point I'm making. (29)

The risk to individual and collective well‐being through a limited sense of belonging was felt strongly in the accounts. The counter to this was when examples were shared of group belonging and finding non‐judgemental spaces: ‘it makes me so happy that I'm finally going to be in an environment where I'm not the odd one out’ (07). Lack of belonging compounds the many losses people are dealing with.

#### 3.2.2 Society and culture: How individuals understand societal value

Participants spoke in their accounts about how society valued them as citizens (see Table 4). Where messages conflicted with aspects of themselves that they valued, there could be profound impacts on mental health. Some people drew a direct link between the amount of money they were able to earn and the extent to which they felt society saw them as valuable, while others spoke more indirectly about how their skills and talents were encouraged or discouraged. A particularly strong example of this was found in the accounts of people from racialised communities. The following example from a young woman who was a professional dancer demonstrates how her attempts to ‘do well’ within predominantly white institutions led to a sense of disorientation and alienation from dance traditions that were important to her.

I began dancing training in London in mixed areas and you could kind of do what you wanted, all was fine. I went to university in a predominantly white area and was the only black female on the course and I definitely felt my, I guess I'm not going to say blackness but my differences were shunned there. […] in choreography, in my university experience, if I came in with dance hall movements or afro movements or house movements, it was just like, ‘That's not what we're asking for’. It was a fight. (21)

This participant spoke of how through her training she became ‘whitewashed’, and that when she was later looking for jobs in Black‐led dance companies, she found that she felt she did not fit there. This sense of dislocation resulted in her feeling like she had lost something essential to herself, with impacts on her mental health.

My experience of inequality through the dance industry and how that has impacted my mental wellbeing, I think it's changed me as a human being. As I said, at first it was about losing my movement. I don't think I ever really got a grasp on that, and I think it became a bit more of losing myself. (21)

However, she also talked about receiving support in the form of studio space and advice from a dance company, and how she felt that reclaiming the dance movement that had been ‘whitewashed’ out of her during her training was a route back to better mental health.

Fast forward to last year, I applied for a job […] I didn't get the job, but they spoke to me and said that they really liked me, and they gave me the space to use their studios and sort of grow and develop as an artist. But within that this woman, it might be just because of the woman, she gave me more information about how to access jobs, where jobs are, where to get training than I've received in my ten years of being under different dance leaders. Now I'm really trying to return to that Afro Caribbean diasporic movement. I'm really trying to return to it, really trying to find it. (21)

How society collectively treats people experiencing severe hardship was also evidenced in the account of a homeless woman. She reported her difficult experiences: ‘I've been told that everybody who begs is a drug addict’ (20). She faced a lot of judgemental attitudes:

This is what I get all the time, I get called a prostitute, a drug addict, ‘Get a life’. People say, ‘You can't be homeless, you're not dirty enough’. Just constant abuse. (20)

A core facet of dealing with experiences of inequality and mental health is stereotyping, underpinned by widely held negative judgements and erroneous beliefs. We heard from a schoolteacher whose workplace did not understand the challenges she faced:

… think one of the first things the head teacher said to me about it was something along the lines of don't get yourself into any dangerous situations or something like, and it was very like victim‐blamey, and I was like this guy clearly has no idea what he's talking about. (22)

These examples build a picture of how inequalities can drive social exclusion, and that related societal attitudes and values can impact mental health. Finding a way back into society can become a key recovery goal: ‘I want to get back into society and it's the only way for me’ (19).

### 3.3 Moving forwards—addressing long‐standing inequality and poor public mental health

In this final section, we explore two dimensions to solutions from participants. The first deals with the Government and institutions that have the power to address inequalities and poor mental health. The second are reflections from participants on personal resilience and coping strategies.

#### 3.3.1 Structural and system‐level responsibilities

The Government, and the systems and structures they preside over, were viewed overall as failing the public: ‘I think the whole system needs to be looked at again and it's very difficult because once the rot has set in you can't change everything in one go’ (28). There was awareness this required political and philosophical overhaul, as well as pragmatic solutions that were ‘bottom up instead of up down' because ‘We have to be very pragmatic. We can't repeat. Many people now are talking about inequality. Many. Yes, but I see, the way I see it is empty shell’ (15, interpreter). This sense of inequality being on the agenda but not meaningfully being addressed was shared by other participants. There was also a great deal of scepticism that people in positions of power were capable of addressing inequalities.

Despite these reservations, local and national governments were viewed as responsible for creating conditions to support better equality and mental health both through policy and legislation and practical support systems. We heard about ‘what the government could do’ (07) to provide better mental health crisis care. How ‘some people have too much and other people too little’ (06) which necessitates rethinking our current economic system to uphold the principles of basic needs outlined as a Human Right. There was a sense of urgency since the Covid‐19 pandemic that significant issues have to be tackled including through education: ‘I mean the government could be doing a lot more’ (02). Education in primary and secondary school was viewed as an important change agent.

We can teach them all these skills at a very early stage then throughout their lives they'll respect everybody else and therefore there will be less problems for others and I think that's going to change the world. (24)

#### 3.3.2 Coping and resilience

While the accounts we heard gave strong examples of how experiences of inequality had a hugely detrimental effect, there were also examples of how people adapted or ‘coped’ and showed resilience. Specific coping mechanisms included the use of therapy, the benefits of pets and friendships and setting work or education goals. In some cases, people demonstrated personal growth.

The things that I've experienced have made me very angry at the world but instead of having a massive tantrum about it, it's just like, ‘Screw you, I'm going to prove you wrong. I'm going to do whatever I want to do’. (02)

We heard in a number of accounts how people who experienced discrimination in the workplace or problems with their housing reached out to find allies that could help them rectify or improve their situations. Particular examples included working with local MPs who could bring about rapid change:

She [my mum] got a really bad blood infection, skin infection because she had to clean off the mould from the walls […] as soon as I emailed the MP, a couple of weeks later, they gave us a permanent home. (04)

Participants spoke to us of finding ways to contribute to their local communities or working in professions where they helped other people. Many spoke of drawing value from those activities.

I'm trying to change my career into using meditation and breath work, Reiki, which is a form of energy healing, and using sound as medicine to heal people, so I think following this passion to try and do something as a career has a really positive effect then on me as well. (26)

Some participants had made peace with the idea that ‘life was unfair’ to be able to function and move on with their lives.

Yes, I've accepted it, it is just life, I can't change it, what I started to do recently is things in your life that you can't change, there's no point being upset about them because it just negatively affects you. So, I surrender to them and move on and just do me essentially. (27)

## 4 DISCUSSION

People who took part in our research were public experts, knowledgeable about both inequalities and mental health. Working with the peer researchers, the conversations generated detailed accounts of inequalities viewed as unjust, personal suffering and part of everyday life. Participants were sensitive to the complexity of talking about mental health and inequalities intersecting with gendered and race‐based experiences in particular; only 10% of the sample were White British. With inequalities, often multiple layers of different overlapping events were shared stretching back several decades, supporting a social determinants of health model.^17^ The circular interaction of inequalities and mental health was understood with financial, housing, immigration and healthcare problems exacerbating poor mental health with racism, gender‐based violence and job loss as contributing factors. Some spoke more as witnesses, observing everyday life unfairness and the State's role in sustaining inequality, while others' accounts were rooted in their own lives and personal traumatic events. Multiple layers of inequality and poor mental health had many impacts; central was the loss of life opportunities. This is consistent with reviews of health inequalities that emphasise the necessity of combatting poverty of opportunity.^1^, ^13^

We observed that as people experienced more inequalities, they were increasingly likely to face struggles and experience discrimination and trauma in their day‐to‐day lives. Being unable to improve their personal circumstances placed additional stress on individuals and, in some cases, resulted in diagnosable mental health problems, along with losses of income, housing and personal relationships that further compounded poor mental health. At the same time, people had difficulty accessing resources (time, money, energy, appropriate local services and support) that would allow them to recover from these difficult experiences. Some individuals spoke of the additional labour they needed to complete tasks that were not required of individuals who did not face the same inequalities as they did, such as navigating the health and social care system as an immigrant with little spoken English. In turn, this reduced the amount of time and energy they had to look after their own physical and mental health.

Our research complements other work in this field. Work in North‐East England surfaced complexity in narratives, including resistance to stereotyping.^28^ Smith and Anderson^30^ built a simplified model from lay accounts of socioeconomic health inequalities with amplifying factors, balanced alongside resilience or resistance factors. We noted resilience in the narratives, including the attitudes people held that they would not be ground down or beaten by circumstances, alongside specific coping mechanisms. There were a range of differing and highly personal strategies to address suffering and injustice. There have also been calls for researchers to do more, assisting healthcare leaders tackle inequalities by producing more practical and relevant work using nonstigmatising language.^7^ Peer researchers in this study reflected on the relative lack of radical political discourse in narratives and our plans to disseminate, without which the status quo remains unchallenged.

### 4.1 Study limitations

This project was planned as a peer‐led project and was delivered by people with experience of poor mental health and structural inequalities until the final analysis and write‐up stage. The write‐up has not been peer‐led due to staff changes and time constraints, which is a limitation, but we maintained peer co‐authorship and input. The focus on experiences generated free‐ranging conversations; however, these could have been underpinned by more conceptual and theoretical work in study design and analysis phases, as well as more emphasis on place‐based differences (Harrow and Lambeth). We also plan additional outputs, including a public summary report and events organised with the community in our study sites, drawing upon the photos provided by participants. During a feedback session we held for the people we interviewed, one participant expressed frustration that we had focused on producing the academic output first and that they had not had the opportunity to participate in the development of community outputs. We acknowledge this criticism. We would have liked to have invited participants back to help us interpret some of their accounts for this paper, but we lacked the capacity to do this effectively. Our study's strengths include a diversity of participants by gender, age and ethnicity spread, a transparent and systematic analysis process and the detailed accounts provided.

### 4.2 Policy recommendations

Our participant accounts suggest changes in policy are needed, to address entrenched problems linking experiences of inequality and poor mental health. The recommendations are based on public experience and detailed knowledge of the consequences of inaction. They include:

1. Healthcare providers listen to the recommendations of mental health service users to co‐design new services and support that address concerns about access to support, quality of care and lack of understanding of individual needs.
2. Education in schools to increase understanding and inclusivity on a range of issues, including mental health stigma, race‐based discrimination, gender‐based violence and LGBTQ+ rights.
3. Better regulation of workplaces to prevent employment discrimination including racism through mandatory accredited training programmes for all staff, establishing mentoring programmes; enforcement of The Equality Act^41^ through clear channels to raise concerns.
4. Improved access to health and social care information so people can better understand and navigate services and how to access systems of mental health support; this is of particular importance for people moving to the United Kingdom from overseas who may find systems and processes very confusing.
5. Better public awareness of the additional work that marginalised groups have to do to get by in everyday life, impacting mental health, from finding suitable products on shop shelves to feeling safe walking down the street.

A key stress test of research recommendations on inequalities is whether they are actionable at a local level.^18^ It is well documented that there are no singular policy solutions, but what is required are upstream preventative work, community‐rooted interventions and support, and keen attention to the agency of individuals themselves in resisting the stigma associated with socioeconomic inequality.^30^ The choice to intervene or not with resources and commitment is known to be political and ideological,^42^ and here we call on political actors to engage with our recommendations above.

## 5 CONCLUSION

The United Kingdom has significant inequality and mental health problems, and people experience these inequalities at a personal level as an embedded part of everyday life and entrenched in broad societal and cultural structures. At the heart of these patterns are individuals facing significant adversity, distress, suffering and loss of opportunities. Key to improving public mental health is a policy change to address social and economic inequalities. New strategies must be found that involve communities, building on our collective knowledge base, to produce positive action and policy reform, to combat living in an unequal society if we are to genuinely impact population mental health.

## AUTHOR CONTRIBUTIONS

Vanessa Pinfold lead for SPHR public mental health programme at McPin, supported the design and development of a peer‐led study alongside Laura E. Fischer and the peer researcher team, worked on ethics application, was a member of the project team attending team meetings and not involved directly in data collection but did support all analysis including coding manuscripts and led on the write‐up of this peer review paper. Rose Thompson supported this study as line manager to Alex Lewington at McPin, was a member of the project team attending team meetings, was providing mentoring support to peer researchers, was not involved directly in data collection but did support this process, supported all analysis including coding manuscripts, led on the write‐up of the study report in parallel to this paper, contributed to sections of this paper including results and discussion sections and reviewed all manuscripts. Alex Lewington, a project co‐ordinator for the study at McPin, worked on ethics application, led the recruitment of participants and data collection, was involved in every interview, coordinated the collective analysis process, created Excel coding files based on team decisions, allocated interviews to the team to code and coded manuscripts and left the team before write‐up but reviewed the draft of this peer review paper. Gillian Samuel is a peer researcher on the study, was involved in the design and development of the peer‐led study, worked on the ethics application, supported the recruitment of participants using their own contacts and community networks, co‐interviewed four people as part of data collection, contributed to analysis process in team meetings, coded interview transcripts and supported the writing of this peer review paper including methods and results sections and reviewed all manuscript drafts of this paper. Sandra Jayacodi is a peer researcher on the study, supported the recruitment of participants using her own contacts and community networks, co‐interviewed 13 people as part of data collection, contributed to the analysis process in team meetings and reviewed all manuscript drafts of this paper. Oliver Jones is a peer researcher on the study, was involved in the design and development of the peer‐led study, worked on the ethics application, supported the recruitment of participants using their own contacts and community networks, co‐interviewed five people as part of data collection, contributed to analysis process in team meetings, supported the writing of this peer review paper including results and discussion sections and reviewed all manuscript drafts of this paper. Ami Vadgama is a peer researcher on the study, was involved in the design and development of a peer‐led study, supported the recruitment of participants using their own contacts and community networks, co‐interviewed two people as part of data collection, contributed to the analysis process in team meetings and reviewed the draft of this peer review paper. Achille Crawford is a peer researcher on the study, supported the recruitment of participants using their own contacts and community networks, co‐interviewed four people as part of data collection, contributed to the analysis process in team meetings and coding interview transcripts and reviewed all manuscript drafts of this paper. Laura E. Fischer was a project coordinator for SPHR public mental health programme at McPin when we designed this study, worked with the team to conceptualise and gain funding for the study, left the team before we began working on the ethics application, delivered trauma‐informed training to the peer researcher team before data collection and supported the drafting of this paper through feedback and revision of manuscripts. Jennifer Dykxhoorn is an academic advisor on the study team, involved in the design of the project including the ethics protocol, attended wider team meetings with all academic advisors to support the delivery of the project and supported the drafting of this paper through feedback and revision of manuscripts. Judi Kidger is an academic advisor on the study team, involved in the design of the project including ethics protocol, attended wider team meetings with all academic advisors to support the delivery of the project and supported the drafting of this paper through feedback and revision of manuscripts. Emily J. Oliver is an academic advisor on the study team, involved in the design of the project including the ethics protocol, attended wider team meetings with all academic advisors to support the delivery of the project and supported the drafting of this paper through feedback and revision of manuscripts. Fiona Duncan is an academic advisor on the study team, supported the ethics application that was submitted at Durham University, attended wider team meetings with all academic advisors to support the delivery of the project and supported the drafting of this paper through feedback and revision of manuscripts.

## CONFLICT OF INTEREST STATEMENT

The authors declare no conflicts of interest.

## ETHICS STATEMENT

Ethical approval was gained from the Durham University Research Ethics Committee (REC): SPORT‐2021‐04‐29T14_45_55‐lxkc61.

## Supporting information

Supporting information.

Supporting information.

Supporting information.

## Data Availability

The data that support the findings of this study are available from the corresponding author upon reasonable request.
