# Supplementary material for: Public perspectives on inequality and mental health: A peer research study
Source: Health Expect. 2023 Oct 2;27(1):e13868. doi: 10.1111/hex.13868 (PMC10768865; doi:10.1111/hex.13868)
Supplement: Supplementary file 1 — Supporting information. [file HEX-27-e13868-s002.docx]

**Inequality and mental health interview topic guide (v2)**

**Question schedule (1-hour):**

In this guide ‘X’ represents a core theme identified from the photos with participants. Depending on the time available 1-3 core themes will be explored in the interview, going at the pace of the interviewee and giving them time to reflect on the questions and their experiences. The questions are only a framework. We will follow the participant exploring what inequality and mental health means to them.

**Part one:**

**Q1:** What does inequality mean to you?

**Q2:** When we talk about mental health what does that term mean to you?

**Q3:** How do you feel inequality or unfairness impacts mental health?

**Part two:**

**Q4:** In your photos you capture X **.** Can you tell me more about why you included X to explore inequality and mental health?

**Q5:** Can you tell me something of your own experiences of [in own words or summary from response]?

**Q6:** How do you think X might relate to your mental health, or to other people’s mental health?

**Q7:** What do people not know about the experiences of X that you think they should?

**Q8:** What needs to change to reduce X?

**Q9:** We have spent some time talking about X, but I can see that Y is also present in your creative work. Why did you include Y?

[repeat above questions 4-8]

**Part three:**

**Q10:** How has covid impacted on your experiences of inequality and or mental health?

**Q11:** Do you have ideas about how the problem X, Y, Z could be remedied?

**Q12:** Who has the power, or whose responsibility is it to change X, Y, Z?
