# Supplementary material for: Public perspectives on inequality and mental health: A peer research study
Source: Health Expect. 2023 Oct 2;27(1):e13868. doi: 10.1111/hex.13868 (PMC10768865; doi:10.1111/hex.13868)

**Appendix 2: Coding process**

There were several layers in the coding process. The first considered factors


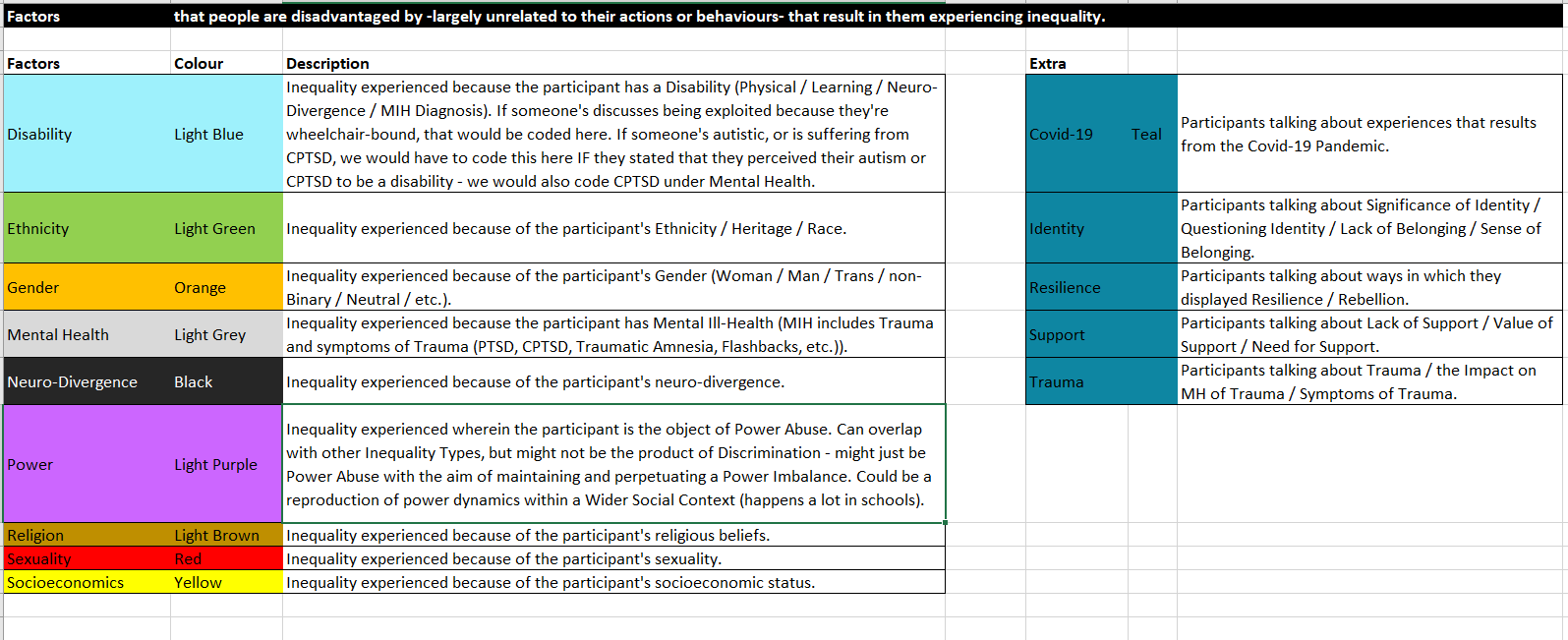


The second layer was context.


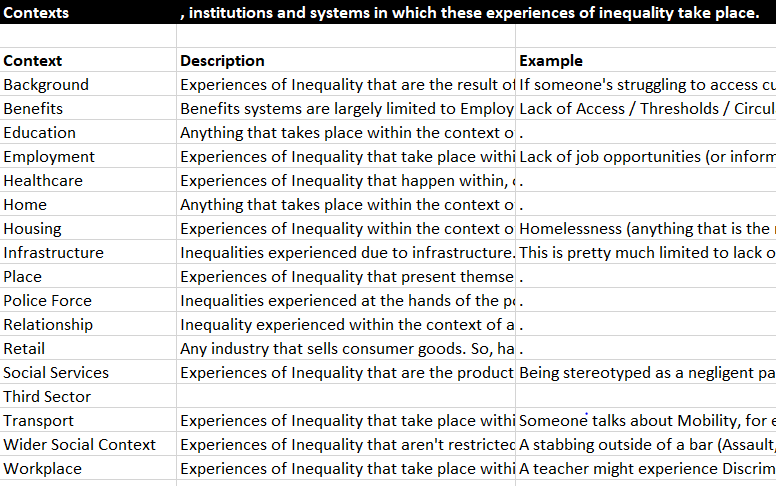


The final layer was ‘manifestation’ which described experiences of inequality.


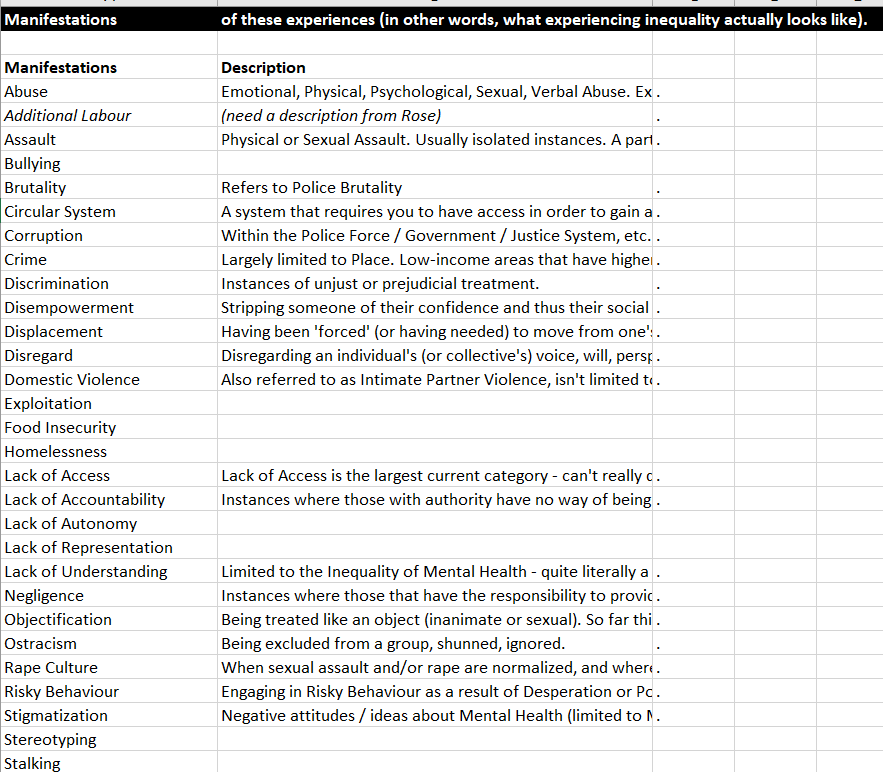


For each transcript we coded segments of text for factor, context, manifestation and impact linking each statement to page number on the transcript. This was done by transferring transcript segments into our excel file coding book. An example is provided below:


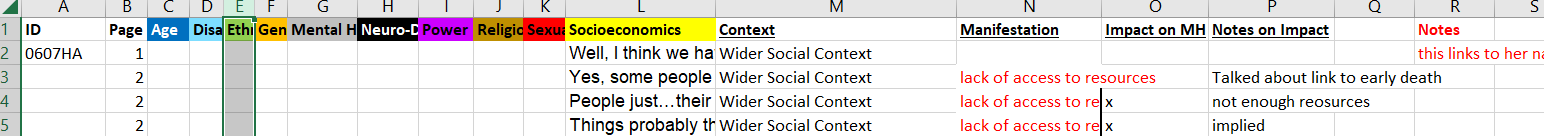


We also coded how participants defined inequalities, how they described mental health and the perceived links again with each extract tagged backed to transcript identification (ID) and page number.


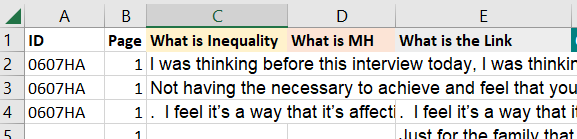


Finally, we also coded extra factors such as impact of covid-19 and coping strategies.


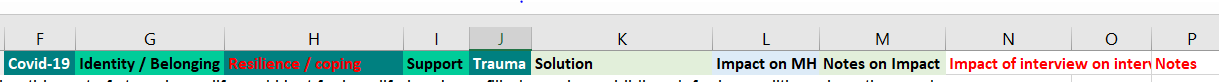

Supplement: Supplementary file 2 — Supporting information. [file HEX-27-e13868-s001.docx]
